# Functional Modulators of Resilience to Age-Associated Dopaminergic Neurodegeneration in C. elegans

**DOI:** 10.1093/geroni/igaa057.2635

**Published:** 2020-12-16

**Authors:** Guy Caldwell

**Affiliations:** The University of Alabama, Tuscaloosa, Alabama, United States

## Abstract

The societal costs of neurodegenerative diseases demand a sense of urgency and innovative strategies toward therapeutic intervention. We have exploited the experimental attributes of roundworm, C. elegans, as a model to expedite discovery of genetic and chemical modifiers of the age-dependent progressive neurodegeneration of dopaminergic neurons commonly associated with Parkinson’s disease (PD). Our model of transgenic nematodes overexpressing the misfolding-prone familial PD gene product, alpha-synuclein, has proven prescient in illuminating key aspects of PD pathology, including vesicular trafficking, autophagy, stress response, lipid dynamics, as well as the first evidence of endolysosomal function in effecting dopaminergic neurodegeneration. Subsequent chemical-genetic analyses reveal a mechanism whereby dopamine transport intersects with epigenetic control of gene expression through proteins functioning in endocytic regulation to modulate neuroprotection. We theorize this reflects a “tunable” nexus of gene-by-environmental regulation where dopamine levels, which impact alpha-synuclein misfolding, are coordinately controlled with epigenetic response to mediate resilience to age-dependent neurodegeneration.

